# Effects of repetitive functional magnetic stimulation in the sacral nerve in patients with neurogenic detrusor overactivity after suprasacral spinal cord injury: a study protocol for a randomized controlled trial

**DOI:** 10.1186/s13063-023-07207-1

**Published:** 2023-03-17

**Authors:** Jiyang Li, Jianxiong Wang, Yue Hu, Rui Jian, Yulu Zhao, Dan Li, Tenggang Wan, Wuga Jike, Fangyuan Xu, Maomao Huang

**Affiliations:** Rehabilitation Medicine Department, The Affiliated Hospital of Southwest Medical University, Luzhou, Sichuan People’s Republic of China

**Keywords:** Neurogenic detrusor overactivity, Repetitive functional magnetic stimulation, Spinal cord injury, Bladder function, Physiotherapy, Randomized controlled trial

## Abstract

**Background:**

Neurogenic detrusor overactivity (NDO) is a serious and common complication after spinal cord injury, affecting patients’ quality of life seriously. Therefore, we developed this research protocol to evaluate the efficacy of repetitive functional magnetic stimulation (rFMS) in the sacral nerve in patients with neurogenic detrusor overactivity (NDO) after suprasacral spinal cord injury (SCI) and provide more options for rFMS in treating NDO after suprasacral SCI.

**Methods:**

This study is a single-center, randomized, parallel-group clinical trial. We will recruit the patients with NDO after suprasacral SCI in the Rehabilitation Department of the Affiliated Hospital of Southwest Medical University from September 2022 to August 2023. They will be assigned to the rFMS group and the sham stimulation group randomly. The sample size is 66, with 33 patients in each group. The rFMS group will receive real rFMS treatment of the sacral nerve (100% stimulation intensity, 5 Hz, 20 min each time, five times a week), and the sham group will receive sham stimulation. Both groups will receive similar treatment strategies, including medication, standard urine management, acupuncture treatment, and health education. The bladder compliance (bladder capacity/detrusor pressure) and pudendal nerve electromyography will be evaluated at baseline, 8th week of treatment. The residual volume of the bladder and bladder diary will be recorded once a week during 8 weeks of treatments. SCI-QOL and NBSS will be evaluated at baseline, the 4th and 8th week of treatment. In addition, the above assessments will be followed up at 8 weeks after the end of treatment.

**Discussion:**

It is expected that the bladder function, symptoms, and quality of life might be significantly improved after rFMS of the sacral nerve.

**Trial registration:**

The China Clinical Trials Registry has approved this study, registration number: ChiCTR2100045148. Registered on April 7, 2021.

**Supplementary Information:**

The online version contains supplementary material available at 10.1186/s13063-023-07207-1.

## Administrative information

Note: the numbers in curly brackets in this protocol refer to SPIRIT checklist item numbers. The order of the items has been modified to group similar items (see http://www.equator-network.org/reporting-guidelines/spirit-2013-statement-defining-standard-protocol-items-for-clinical-trials/).

**Table:** 

Title (1)	Effects of repetitive functional magnetic stimulation in the sacral nerve in patients with neurogenic detrusor overactivity after suprasacral spinal cord injury: a study protocol for a randomized controlled trial.
Trial registration {2a and 2b}.	The China Clinical Trials Registry has approved this study, registration number: ChiCTR2100045148.
Protocol version (2)	Version 2 of 26–10-2022
Funding (2)	This work will be supported by School-level scientific research project of Southwest Medical University No. 2021ZKQN042, Southwest Medical University Youth Science and Technology Talents Cultivation Program No. 00160273, Sichuan Provincial Cadre Health Research Project No. chuanganyan 2021–1505. Funders have no role in study design, data collection and analysis, and manuscript writing.
Author details (3)	Rehabilitation Medicine Department, The Affiliated Hospital of Southwest Medical University, Luzhou, Sichuan, People’s Republic of China.
Name and contact information for the trial sponsor {5b}	Jiyang Li, MSc. E-mail: 849215633@qq.com Jianxiong Wang, Ph.D. E-mail: jianxiongwang_swmu@126.com Yue Hu, MSc. Email: 547955626@qq.com Rui Jian, MSc. Email: 13982750867@163.com Yulu Zhao, B.S. E-mail:563144343@qq.com Dan Li, MSc. E-mail: 947037100@qq.com Tenggang Wan, B.S. E-mail: 350303280@qq.com Wuga Jike, B.S. E-mail: 1772692885@qq.com Fangyuan Xu, MSc. ** E-mail: x5144@163.com Maomao Huang, MSc. ** E-mail: 973342240@qq.com.
Role of sponsor {5c}	JL and MH designed the protocol and drafted the manuscript. J-XW, FX and YH received project funding and consulted during the research. RJ and DL are physicians recruiting patients. TW is a physical therapist who uses rFMS to treat patients with NDO. YZ is responsible for baseline assessment and follow-up. JW is a research assistant for the project and is responsible for data collection and analysis. All authors have commented on drafts of this paper and have read and approved this final manuscript.

## Introduction

### Background and rationale {6a}

Neurogenic detrusor overactivity (NDO) is recognized when detrusor overactivity accompanies a relevant neurological condition [1]. As a type of neurogenic bladder (NB), NDO is quite common in patients with suprasacral spinal cord injury (SCI). NDO can lead to intermittent increases in intravesical pressure and urine leakage [2]. Untreated NDO can lead to many complications, such as recurrent urinary tract infections, urinary calculi, and urethral strictures, which seriously affect the patient’s quality of life [3]. Currently, interventions for NDO include medicine, acupuncture, sacral neuromodulation, peripheral tibial nerve stimulation, pelvic floor muscle function training, and surgical intervention [4–6]. Functional magnetic stimulation (FMS) is a non-invasive treatment for NDO that has emerged in recent years. Investigators have observed a well-tolerated treatment of stress urinary incontinence with no pain or side effects by placing the therapeutic coils flatly on the sacrum (S2–S4), resulting in an electromagnetic impact [7]. Previous studies have shown that magnetic stimulation might effectively treat NB [5, 8, 9]. However, these articles were observational studies or small sample studies. There is a lack of high-quality research on repetitive functional magnetic stimulation (rFMS) of sacral nerves in the treatment of NDO secondary to suprasacral SCI. Therefore, we developed this research to evaluate the efficacy of rFMS in the sacral nerve in patients with NDO after suprasacral SCI and provide more options for rFMS in treating NDO.

### Objectives {7}

1. To evaluate the efficacy of repetitive functional magnetic stimulation (rFMS) in the sacral nerve in patients with neurogenic detrusor overactivity (NDO) after suprasacral spinal cord injury (SCI)
2. To provide more options for rFMS in treating NDO after suprasacral SCI.

### Trial design {8}

**Fig. 1:**
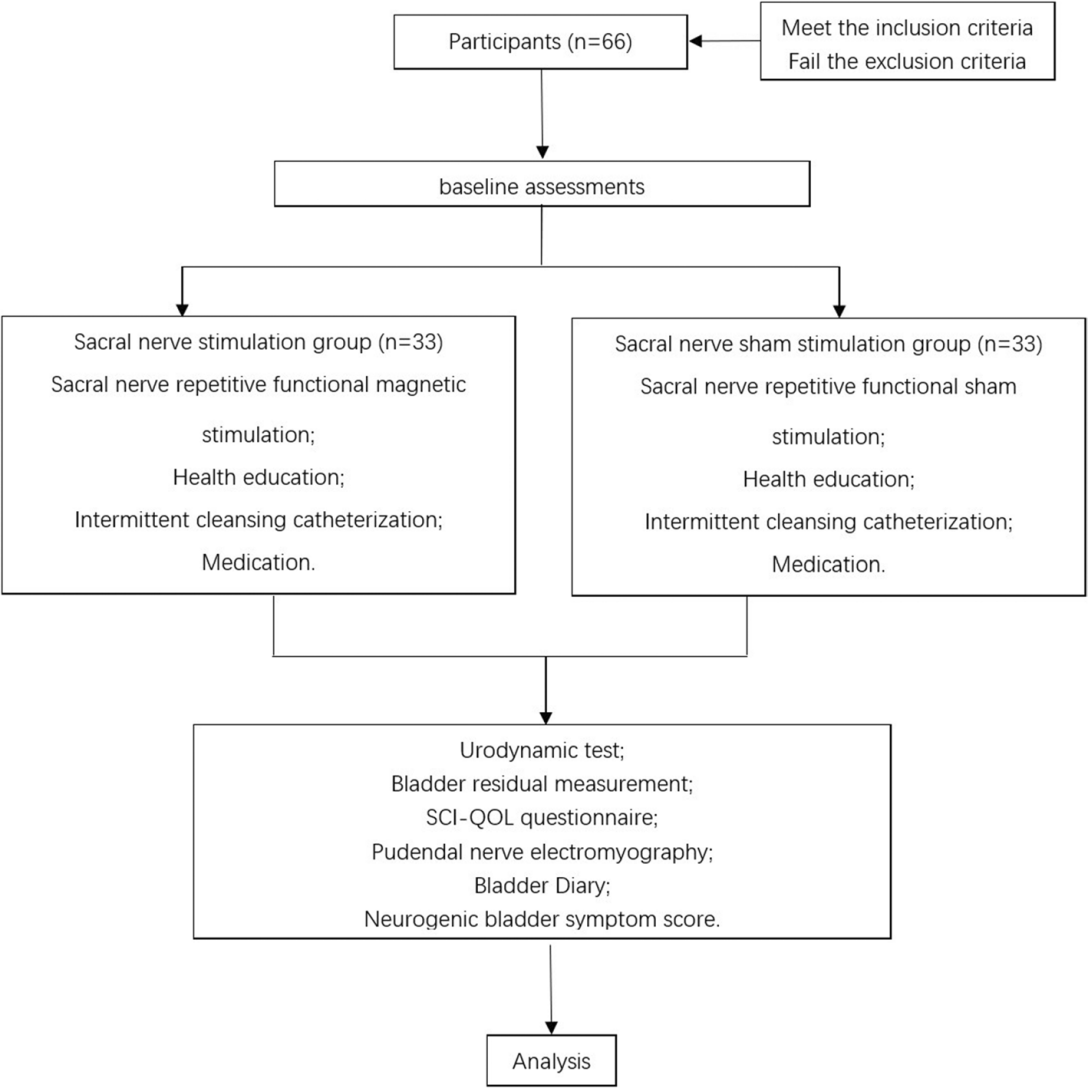
Summary of study protocol

This study will be a double-blind, randomized controlled trial. Patients who fulfill the inclusion criteria and sign the informed consent form will be randomly divided into the rFMS group and sham rFMS group (sham group) in a 1:1 ratio. Assessments are made before treatment, during 8 weeks treatment, and 8 weeks after treatment (Fig. 1). The purpose of this study is to design a high-quality randomized controlled trial and realize the complete randomization of participants. The patients, professional evaluators, and data analysts are all blinded, so as to provide reliable data for the recovery of bladder function of suprasacral SCI with NDO after rFMS treatment.

## Methods: participants, interventions, and outcomes

### Study setting {9}

All the patients will come from the Rehabilitation Medicine Department, the Affiliated Hospital of Southwest Medical University. Patients are considered for inclusion if they meet the criteria as defined below.

### Eligibility criteria {10}

The inclusion criteria are as follows: ① definitely diagnosed as suprasacral SCI [10], the course of the disease is more than one month; ② age: 18–60 years old; ③ urodynamics indicates NDO [11]. The exclusion criteria are as follows: ① patients with metal implants in the lumbosacral area; ② patients with cognitive dysfunction who cannot cooperate; ③ patients with unstable vital signs; ④ those who have a history of botulinum toxin injection within 6 months or a history of bladder/sphincter surgery; ⑤ patients with unstable fractures who cannot complete urodynamics. Once patients are enrolled, we will use questionnaires to record participants’ baseline data such as age, gender, symptoms, duration, past treatments (including medications and rehabilitation), and more.

### Who will take informed consent? {26a}

Patients with NDO will be screened for eligibility to participate in this study based on the abovementioned criteria. patients assessed as qualified by the treatment group are invited to meet with the research physician to discuss any remaining questions and sign the informed consent.

### Additional consent provisions for collection and use of participant data and biological specimens {26b}

In the consent form, participants will be asked if they agree to use of their data when they choose to withdraw from the trial. Participants will also be asked for permission for the research team to share relevant data with people from the Universities taking part in the research or from regulatory authorities, where relevant. This trial does not involve collecting biological specimens for storage.

## Interventions

### Explanation for the choice of comparators {6b}

The sham group will receive sham rFMS stimulation and medication, standard urine management, acupuncture treatment, and health education.

### Intervention description {11a}

The rFMS group will receive real rFMS treatment of the sacral nerve, while the sham group will receive sham stimulation. Both groups will receive similar treatment strategies, including medication, standard urine management, acupuncture treatment, and health education.

#### rFMS and sham stimulation

All magnetic stimulation treatments will be performed by an experienced rehabilitation therapist specializing in neurorehabilitation for over 5 years. For the rFMS group, the patient takes the prone position and aligns the centers of the two circular magnetic coils with the third sacral nerve (S3) holes on both sides (the upper edge of the sacrum and the midpoint of the coccyx connect one lateral finger on the left and right sides). For the first treatment, motor thresholds will be measured with single-pulse stimulation. The parameters of rFMS treatment will be as follows: the stimulation intensity will be 100%; the frequency will be 5 Hz; 20 min each time, five times a week, for 8 consecutive weeks. Simultaneously, the sham group will use a sham coil, which will not generate a magnetic field but has a similar appearance and sound during operation. The frequency and treatment time will be the same as in the rFMS group, except that the coil does not generate a magnetic field.

#### Medication

According to relevant guidelines [12], each patient will receive tolterodine with a dose of 2 mg twice a day.

#### Bladder management strategy

For bladder emptying, the individualized management strategy is selected according to the patient’s condition, mainly including indwelling catheterization and intermittent catheterization, with the frequency determined according to residual urine.

#### Acupuncture treatment

Acupuncture treatment will be performed by an experienced traditional Chinese medicine doctor who has been in acupuncture for more than 5 years. Select Tianshu, Guanyuan, Qihai, Sanyinjiao, Yinlingquan, and other acupoints to clean and disinfect each point; then acupuncture the acupoints, and leave the needle for 30 min. The acupuncture will be conducted once a day, five times a week, for 8 weeks.

### Criteria for discontinuing or modifying allocated interventions {11b}

There will be no pre-defined criteria for stopping or modifying the allocated intervention. Participation is voluntary. Participants may withdraw from the voluntary trial at any time for any specific reason. Withdrawal from participation has no negative consequences or adverse effects on participants. Researchers may also terminate participation if serious adverse events occur during the intervention. We will suggest that patients who withdraw from the trial continue to standardize the treatment of NDO, so that their subsequent normal medical care will not be affected.

### Strategies to improve adherence to interventions {11c}

Encourage adherence to treatment through weekly evaluations and psychological intervention by experienced psychologists when necessary. Strategies may include encouragement and setting specific goals and dates for weekly physical and drug therapy. The whole process of rFMS treatment will be supervised by an experienced rehabilitation therapist specializing in neurorehabilitation in our center.

### Relevant concomitant care permitted or prohibited during the trial {11d}

All participants will receive medication, standard urine management, acupuncture treatment, and health education. Health education is an organized, planned, and implemented educational activity. It assists individuals and groups in acquiring knowledge about health care, establishing health ideas, and voluntarily embracing educational activities supportive of healthy habits and lifestyles through information dissemination and behavioral intervention. Health education about NDO includes the rationale for bladder emptying options, drinking water plans, prevention of urinary tract infections, etc.

### Provisions for post-trial care {30}

In the event of study-related damage or injuries, the Affiliated Hospital of Southwest Medical University shall provide free symptomatic treatment. If the injury is serious, invite relevant experts for treatment and provide medical compensation.

### Outcomes {12}

#### Primary outcome

The primary outcome is the bladder compliance measured by urodynamic testing. Bladder compliance is the relationship between a change in bladder capacity and a change in detrusor pressure (Pdet) [13]. Urodynamic examinations will be performed by the same experienced technician who has been performing urodynamic examinations for more than 5 years. The detection steps are as follows: ① The day before the examination, the patient is given a glycerin enema to clear up dry stools. The skin around the anus and perineum is prepared, and pubic hair is removed if necessary. ② On the day of the examination, the patient is placed in a supine position, the genital area is routinely disinfected, sterile towels are applied, and a 6F double-lumen manometry catheter is inserted into the bladder through the urethra and will be fixed with tape (penis for males, perineal for females). Insert an 8F balloon catheter into the rectum, inject an appropriate amount of fluid into the balloon of the rectal catheter, and place it in the patient’s rectum to a depth of about 10 cm. ③ During the measurement, the patient will take the lithotomy position. And the normal saline will fill the bladder with a perfusion rate of 50 ml/min by using a water pump. The patient will be told to cough before the start of perfusion to suppress the urge to urinate during perfusion, to cough again before the end of the assay, and to report when the patient feels the first urge to urinate and feels that the maximum cystometry volume been reached. Measure and record using the sensor-related indicators. ④ Obtain bladder compliance, abdominal pressure (Pabd), intravesical pressure (Pves), Pdet, urine flow rate (Q), and other indicators. Safe bladder capacity refers to the bladder capacity when the bladder pressure reaches 40 cmH_2_O or occurring urine leakage in the filling period [14]. And the Pdet will be measured in the urodynamic tests described above.

#### Secondary outcomes

The secondary outcome indicators include the residual volume of the bladder, the Spinal Cord Injury-Quality of Life, bladder diary, Neurogenic Bladder Symptom Score, and pudendal nerve electromyography. Besides objective indicators, those outcomes can also reflect the effect of rFMS on NDO-associated symptoms and the Spinal Cord Injury-Quality of Life (SCI-QOL).

Participants with partially voluntary voiding function will be taken bladder residual urine measurements immediately after voiding once a week. Each measurement will scan the bladder in the sagittal and transverse planes. The probe is located on the midline above the symphysis pubis to obtain a sagittal view of the lower abdomen. When the largest picture of the bladder appears on the screen, freeze the image and measure the height (h), depth (d), and cross-sectional area (AS) of the bladder. Then place the probe laterally above the symphysis pubis, at right angles to the sagittal plane, and provide another part of the lower abdomen. When the largest picture appears on the screen, freeze the image and measure the width (w), depth (d), and cross-sectional area (AT) of the bladder. According to previous studies [15], the residual volume is approximately equal to the cross-sectional area of the bladder when measured in the sagittal plane multiplied by the width of the bladder.

SCI-QOL measurement system will assess the quality of life. It includes four aspects: physical health, emotional health, social participation, and physical function [16]. Each aspect has 3–6 sub-items, which can comprehensively evaluate patients’ physical and psychological functions with SCI. SCI-QOL will evaluate the patients at the time of evaluation, and the results will be recorded.

The bladder diary will measure the frequency of acute urinary incontinence, which can reflect the frequency of urinary incontinence and quantify bladder symptoms [17]. Patients will record the frequency of acute urinary incontinence weekly for 8 weeks of the entire trial and during follow-up.

The pudendal nerve and subsacral motor neuron involvement are significantly associated with chronic SCI [18]. Electromyogram (EMG) is the muscle bioelectric pattern recorded with an electromyography instrument. It can evaluate the function of nerves. Studies [19] have found that pudendal nerve stimulation can cause reflex bladder contraction in patients with chronic SCI, which indicates that the function of the pudendal nerve is related to bladder function and can be used to evaluate bladder function. In our study, quantitative external anal sphincter (EAS) muscle electromyography (EMG), pudendal nerve terminal motor latency (PNTML) testing, pudendal somatosensory evoked potentials (PSEP), and bulbocavernosus reflex (BCR) will be measured [20]. The resting and light exertion EMG of EAS will be detected by using concentric needle electrodes [21]. The pudendal nerve terminal motor latency will be recorded by finger cuff electrodes [22]. For pudendal nerve PSEP, the P40 latency will be measured by the recording electrode placing in Cz (active electrode), while the stimulation electrode will be placed in the pubic symphysis [23]. For BCR, mean latency values will be recorded by concentric needle electrodes [24]. Specifically, the patient will be first placed in the lithotomy position, and then the BCR will be elicited by rapidly pressing down on the glans with the probe. Electrodes will be placed around the anus and on the inner thighs at the 3 o’clock and 9 o’clock positions to capture the electrical activity generated by this reflex. Pressure from the probe against the glans generates the initial stimulus waveform. This will be followed by a second elevated waveform or contraction in response to the BCR. Latency will be calculated based on the time between these two events.

Neurogenic Bladder Symptom Score (NBSS) evaluates patients’ symptoms with NB from three dimensions urinary incontinence, storage and urination, and outcome. It is an effective and comprehensive tool for assessing NB symptoms in patients with SCI [25]. NBSS includes three dimensions of urinary incontinence, storage, urination, and outcome, with 24 items, of which two are unscored. The first item is classified according to the patient’s bladder management, and the last item is the patient’s bladder management. The overall quality of life assessment assesses the patient’s urination pattern and quality of life. Each item is worth 0 ~ 3 points or 0 ~ 4 points. The scale’s total score is 74 points, with higher scores indicating more obvious NB symptoms.

### Participant timeline {13}

Before starting the intervention, participant baseline data will be collected, as well as bladder capacity and Pdet measured by urodynamic testing, the residual volume of the bladder, quality of life, bladder diary, NBSS, and pudendal nerve electromyography. After that, interventions will be performed five times a week for 8 consecutive weeks. The bladder compliance (bladder capacity/Pdet) and pudendal nerve electromyography will be evaluated at baseline, 8th week of treatment. The residual volume of the bladder and bladder diary will be recorded once a week during 8 weeks of treatments. SCI-QOL and NBSS will be evaluated at baseline, the 4th and 8th week of treatment. In addition, the above assessments will be followed up at 8 weeks after the end of treatment. The participant timeline is presented in Table 1.

**Table 1:** Schedules for follow-up assessments and data collection

		**Study period**					
	**Enrolment**	**Allocation**	**Post-allocation**				**Close-out**
**Timepoint**	*** − t***_***1***_	**0**	***t***_***0***_***: five times a week, for 8 weeks***	***t***_***1***_***: Once a week during 8 weeks of treatments***	***t***_***2***_***: 4th week of treatment***	***t***_***3***_***: 8th week of treatment***	***T***_***4***_***: 8 weeks after the end of treatment***
**Enrolment:**							
**Eligibility screen**	X						
**Informed consent**	X						
**Allocation**		X					
**Interventions:**							
**rFMS and sham stimulation**			X				
**Medication**			X				
**Bladder management strategy**		X					
**Acupuncture treatment**			X				
**Assessments:**							
***Bladder capacity***	X					X	X
***Pdet***	X					X	X
***Bladder compliance***	X					X	X
***Residual volume of the bladder***	X			X			X
***Bladder diary***	X			X			X
***SCI-QOL***	X				X	X	X
***NBSS***	X				X	X	X
***Pudendal nerve electromyography***	X					X	X

### Sample size {14}

Sample size was calculated using bladder compliance as the main outcome. According to the analysis of previous research results [12, 26], bladder compliance change after therapy of 8 ml per cm H_2_O with a standard deviation of 9 ml per cm H_2_O was used in the calculation. Assuming that the standardized effect size is 0.25, the probability of type I error is 5%, and the probability of avoiding type II error is 90%, the trial requires at least 26 participants in each group. Considering the allowable 20% dropout rate, approximately 33 patients per group will eventually be needed.

### Recruitment {15}

We will recruit patients through publication and distribution of posters and leaflets containing information of the study. Most of the patients are from the Department of Rehabilitation Medicine, Affiliated Hospital of Southwest Medical University. If the number of patients in rehabilitation medicine cannot meet the required number of participants in our experiment, we will continue to recruit patients with NDO referred by neurosurgery, urology, and other departments until recruitment is completed. The official start of recruitment was on 01 September 2022.

## Assignment of interventions: allocation

### Sequence generation {16a}

The random allocation table will be generated by SPSS 23.0 software, and the included cases will be randomly divided into treatment and control groups. The research coordinator is responsible for randomization, which will be carried out by simple 1: 1 draw (for each pair of patients who are recruited).

### Concealment mechanism {16b}

The random allocation table will be sealed in duplicate in an opaque envelope and stored in the third party and the main research unit, respectively. After the clinical trial is over and all the case report forms are double-checked by the participating clinical treatment units, evaluation units, and third parties, the data will be finally locked.

### Implementation {16c}

Randomization will be implemented by a research assistant who is not involved in the trial in any form. After ensuring patients fulfill inclusion criteria and informed consent forms are obtained from them, the envelopes are opened by the rehabilitation therapist specializing in rFMS and rFMS treatment is carried out.

## Assignment of interventions: blinding

### Who will be blinded {17a}

All of the trial participants, outcome assessors, and data analysts will be blinded after assignment to interventions. They only know the patient number and they don't know the specific group of patients.

### Procedure for unblinding if needed {17b}

Unblinding will be allowed under emergencies such as the incidence of serious adverse effects.

## Data collection and management

### Plans for assessment and collection of outcomes {18a}

Urodynamic examinations will be performed by the same experienced technician who has been performing urodynamic examinations for more than 5 years. The residual volume of the bladder, the Spinal Cord Injury-Quality of Life, Neurogenic Bladder Symptom Score, and pudendal nerve electromyography will be conducted by professional evaluators. The bladder diary comes from patient records.

All researchers, including the rFMS therapist, outcome assessors, data collectors, data managers, and statisticians, will undergo training before the experiment. All data will be anonymized and saved in an encryption study folder and only the study team has access to this study folder.

### Plans to promote participant retention and complete follow-up {18b}

The importance of completion of the treatment and follow-up assessment will be stressed. Patients are allowed to stop the experiment at any time during the study. If possible, the patient will be asked to complete the follow-up assessments at 8 weeks after the end of treatment. Throughout the study period, the researchers will keep in close contact with the participants if necessary.

### Data management {19}

The epidata (version 4.6) will be used to manage data. After finishing all the assessments, data will be checked by trained personnel and two people will be both processing data to ensure data accuracy.

### Confidentiality {27}

All data will be stored on the local server of the Rehabilitation Department of the Affiliated Hospital of Southwest Medical University. All data, including personal data collected, will be kept strictly confidential.

### Plans for collection, laboratory evaluation, and storage of biological specimens for genetic or molecular analysis in this trial/future use {33}

There are no plans for collection and evaluation of biological specimens in this study.

## Statistical methods

### Statistical methods for primary and secondary outcomes {20a}

We will use SPSS 23.0 statistical software for data analysis with a statistical significance level set at* p* ≤ 0.05. We will apply the chi-square test or Fisher’s exact test to analyze categorical data such as symptoms or gender. Data that is not normally distributed will be analyzed using nonparametric analysis. In addition, repeated measures ANOVA and post hoc tests will be used to analyze statistically significant differences between and within the groups.

### Interim analyses {21b}

No interim analyses are planned.

### Methods for additional analyses (e.g., subgroup analyses) {20b}

No additional analyses are planned.

### Methods in analysis to handle protocol non-adherence and any statistical methods to handle missing data {20c}

In this study, we plan to perform an intention-to-treat analysis (ITT) and a per-protocol analysis (PP). Consistent results will help determine the conclusions of the study. If inconsistent, we will do further analysis and discussion. Furthermore, we will do our best to reduce missing data. We will use a modal mixture model to fill in the missing outcome data if data is missing.

### Plans to give access to the full protocol, participant-level data and statistical code {31c}

The datasets during the current study and statistical code are available from the corresponding author on reasonable request, as is the full protocol.

## Oversight and monitoring

### Composition of the coordinating center and trial steering committee {5d}

The study will be monitored by an independent internal monitor. The direct test group responsible for trial management, routine activities, and intervention management will meet biweekly. The broader team, which includes rehabilitation physicians, rehabilitation nurses, and rehabilitation therapists, will meet every month. The study investigators will be responsible for monitoring and managing data quality, assessing completeness and accuracy of data collection, implementation and adherence to the study protocol, and assessment of outcomes.

### Composition of the data monitoring committee, its role and reporting structure {21a}

Because our single-center trial is small, involved few researchers and has no external funding, there are no need for an additional data monitoring committee.

### Adverse event reporting and harms {22}

Adverse events will be collected and monitored by professionals. Any adverse events that occur during the trial (for example, worsening symptoms and psychological disturbances) will be classified as adverse events. All adverse events will be discussed weekly regarding causality and severity, and further action may be taken based on the results.

### Frequency and plans for auditing trial conduct {23}

The Ethics Committee of Affiliated Hospital of Southwest Medical University is the Trial Steering Committee and will supervise the trial, and the committee will meet every year.

### Plans for communicating important protocol amendments to relevant parties (e.g., trial participants, ethical committees) {25}

If any protocol needs to be modified, the Court’s Ethics Committee will review it again and, upon approval, update the trial registry and protocol. Any violation of the protocol will be fully documented using the Violation Report form.

### Dissemination plans {31a}

The results will be communicated via journal publications or conference presentations. If participants are interested in the results of the trial, they will be notified at the end of the trial.

## Discussion

This study will be a randomized, double-blind, sham-controlled trial to investigate the effect of rFMS in the sacral nerve on NDO after SCI. Bladder function, symptoms, and SCI-QOL are the main observation objects of our study. The intervention and follow-up time will last 8 weeks, respectively, to observe the short-term efficacy of rFMS, which will lay a foundation for further research.

As the most common type of NB, the routine treatment of NDO mainly includes anticholinergic drugs, botulinum toxin injection, acupuncture, and surgical treatments [2, 27]. However, these conventional treatments also have some limitations, including side effects and poor compliance [27]. For example, anticholinergic drugs may produce arrhythmias, visual blurring, xerostomia, and constipation. Botulinum toxin injection might alter the bladder sensory system, which will shorten its effectiveness [28]. Most surgical managements are invasive operations and are only considered in cases of severe bladder contracture due to NDO. Hence, safe nonpharmacological and nonoperative therapies those may be effective in treating NDO have attracted the attention of researchers. In recent years, Non-invasive neuromodulation techniques have emerged as new treatments, such as Repetitive transcranial magnetic stimulation (rTMS), electrical stimulation of peripheral nerves, and FMS of sacral nerves. rTMS represents a potentially valuable treatment for neurogenic urinary disturbances by inducing a long-lasting modulation of spinal cord excitability [29]. Centonze found that 5-Hz rTMS in the motor cortex produces desirable effects on the voiding phase during the micturition cycle in patients with multiple sclerosis, indicating that rTMS enhanced corticospinal tract excitability to facilitate detrusor contraction and/or urethral sphincter relaxation [30]. Some studies showed that transcutaneous tibial nerve stimulation might effectively improve urodynamics parameters and alter the course of SCI with NB via neuromodulation in the short term [31, 32]. It has been shown that repetitive lumbosacral nerve magnetic stimulation at 15 Hz may improve urinary dysfunction secondary to lumbosacral nerve injury [33]. Tetsuyuki Fujishiro found that rFMS of the sacral roots may be helpful in controlling urinary frequency and urge incontinence [34]. Po-Yi Tsai has confirmed the efficacy of 5-Hz sacral FMS in decreasing stress urinary incontinence [35]. Bycroft found that sacral rFMS might inhibit detrusor contraction by “rebound” effect in NDO and had not stimulated preganglionic parasympathetic to induce bladder contractions [6]. But the sample size was too small, and outcome assessment was undetailed in this study. Lamyaa A found that the effects of pulsed electromagnetic field therapy in patients with NDO were better than TENS according to the urodynamics outcome [8]. However, this study only used urodynamics to evaluate the bladder function and did not use blinding. T Yamanishi found that the inhibition of detrusor overactivity appeared more significant in the magnetic stimulation group than in the electrical stimulation group [36]. But the outcome was evaluated only by bladder capacity, and no follow-up was conducted to observe its effect in the research. So, there is a lack of high-quality research on sacral rFMS in treating NDO secondary to suprasacral SCI. And the review suggested that a high-quality randomized controlled trial about neuro-regulation in the treatment of NB might be needed [37]. Thus, we designed a stricter study to explore the efficacy and safety of rFMS in treating NDO with sham rFMS as a comparison. In addition, to the best of our knowledge, this will be the first double-blind study on rFMS to treat NDO. Moreover, we will also use subjective and objective outcomes to obverse its effects.

In terms of outcome evaluation, besides subjective assessment methods such as bladder diary, SCI-QOL questionnaire, and NBSS, we will also use objective assessments, including urodynamics and pudendal nerve electromyography. Urodynamics assessments are the primary basis for evaluating NB, including bladder capacity, urinary flowmetry, bladder cystometry, electromyogram, Valsalva leak point pressure, and detrusor leak point pressure [27]. Video urodynamics combines voiding cystourethrography with multichannel urodynamics to provide the most comprehensive assessment of bladder function in patients with SCI. When vesicoureteral reflux occurs, it is also possible to record the volume and pressure at which the reflux started [38]. Urodynamic diagnoses, such as NDO, impaired compliance, and reduced bladder capacity, can identify a patient with a potentially higher risk of urological complications as one of the essential indicators of urodynamics, the duration of the NDO contraction may also predict renal deterioration [4].

The changes in pudendal innervation are closely related to NB filling symptoms and detrusor overactivity [39]. There are two different pathological mechanisms of NDO secondary to SCI: increased afferent signaling and abnormal handling of efferent activity [28]. The high center lost its inhibitory effect on the low center after suprasacral SCI. Suprasacral SCI releases the spinal cord segmental micturition reflex from supraspinal modulation and increases the injury-induced release of bladder neurotrophic factors such as brain-derived neurotrophic factor, glial‐derived neurotrophic factor, and ciliary neurotrophic factor. These neurotrophic factors promote Na + channel activation and K + channel deactivation, which subsequently increase the hyperexcitability of the dorsal root ganglion neurons innervating the urinary bladder and activate hypermechanosensitive C‐fiber bladder wall afferents [40–42]. rFMS of the sacral nerve can improve NDO symptoms by different pathways. The excitatory impulses produced by magnetic stimulation ascending to the thoracolumbar sympathetic neurons can excite the sympathetic nerve and inhibit the parasympathetic nerve, thus decreasing detrusor pressure [43]. Stimulation of the sacral nerve might induce an interneuronal change in the spinal reflex arc or spinobulbospinal reflex arc to inhibit the activity of the dominant C-fibers in neurological injury [44]. Therefore, it is meaningful to record pudendal nerve evoked potentials. At present, many studies have used urodynamics or electrophysiological examination of the pudendal nerve to evaluate sacral nerve implantation electrodes for regulating the NB [45–48]. This is a clinical trial, and we will not explore its specific mechanism further. But we will also measure the pudendal nerve evoked potential as one of the outcomes. At the same time, NDO will seriously affect patients’ quality of life. Therefore, we will use subjective assessments (bladder diary, SCI-QOL questionnaire, NBSS) and objective assessments (urodynamics, pudendal nerve electromyography) to evaluate the efficacy of sacral nerve rFMS in the treatment of NDO after SCI.

Our study will have some limitations. First, we will only enroll patients with suprasacral SCI, so the conclusion of our study may be suitable for patients with NDO after suprasacral SCI. Other studies are needed to observe the efficacy in treating NB after subsacral SCI. Second, the follow-up of this trial will be only 8 weeks. After 8 weeks of follow-up, we will decide whether to continue the follow-up according to the patients’ wishes.

## Trial status

This publication is based on version 1 of the rFMS protocol dated 01 January 2022. The official start of recruitment was on 01 September 2022. The estimated end date of the trial is 31 August 2023 and recruitment of patients is ongoing.

## Supplementary Information

**Additional file 1. **Reporting checklist for protocol of a clinical trial.

**Additional file 2.** Informed consent form.

## Data Availability

The datasets used or analyzed during the current study are available from the corresponding author on reasonable request.

## Abbreviations

NDO: Neurogenic detrusor overactivity
rFMS: Repetitive functional magnetic stimulation
SCI: Spinal cord injury
SCI-QOL: Spinal Cord Injury-Quality of Life
NBSS: Neurogenic Bladder Symptom Score
Pabd: Abdominal pressure
Pves: Intravesical pressure
Pdet: Detrusor pressure
EAS: External anal sphincter
EMG: Muscle electromyography
PNTML: Pudendal nerve terminal motor latency
PSEP: Pudendal somatosensory evoked potentials
BCR: Bulbocavernosus reflex
