# Supplementary material for: Effects of repetitive functional magnetic stimulation in the sacral nerve in patients with neurogenic detrusor overactivity after suprasacral spinal cord injury: a study protocol for a randomized controlled trial
Source: Trials. 2023 Mar 17;24:199. doi: 10.1186/s13063-023-07207-1 (PMC10022156; doi:10.1186/s13063-023-07207-1)
Supplement: Supplementary file 2 — Additional file 2. Informed consent form. [file 13063_2023_7207_MOESM2_ESM.docx]

**Informed consent form**

Subjects' Instructions page

Protocol Title: Effects of Repetitive Functional Magnetic Stimulation in the sacral nerve in patients with neurogenic detrusor overactivity after suprasacral spinal cord injury: a randomized controlled trial

Principal Investigator: Maomao Huang

Sponsor: Affiliated Hospital of Southwest Medical University

Dear Subjects,

You were invited to participate in a randomized controlled trial of effects of Repetitive Functional Magnetic Stimulation in the sacral nerve in patients with neurogenic detrusor overactivity after suprasacral spinal cord injury, supported by the Affiliated Hospital of Southwest Medical University. Please read this informed consent form carefully and make a careful decision on whether or not to participate in this study. Participating in this study is entirely your choice. As a subject, you must give your written consent before joining a clinical study. When your research doctor or researcher discusses an informed consent form with you, you can ask him/her to explain to you if you don't understand. We encourage you to have a full discussion with your family and friends before making the decision to participate in this research. You have the right to refuse to participate in the Study or to withdraw from the Study at any time without penalty or loss of your rights. If you are enrolled in another study, please let your research doctor or researcher know. The background, purpose, research process and other important information of this study are as follows:

**Research background**

Neurogenic detrusor overactivity is a common complication after spinal cord injury, with neurogenic bladder occurring in approximately 70 to 84 percent of patients after spinal cord injury. Hyperreflexia of the detrusor or detrusor and sphincter dysfunction occurs in 95 percent of patients, making improvement in bladder function the primary research focus in patients with SCI. Numerous researchers have studied the role of pelvic floor muscle training, biofeedback, nerve stimulation, acupuncture, drugs, and surgery in the treatment of neurogenic bladder after spinal cord injury, and short-term treatment has shown efficacy, but long-term efficacy is not obvious. As a treatment for urinary dysfunction, sacral nerve stimulation has been approved by the US FDA to correct urinary dysfunction by exciting or inhibiting S2-4 nerve roots, but this method requires surgical implantation of electrodes, and various surgical complications such as infection, cerebrospinal fluid leakage, nerve root injury, pain, etc., and expensive surgical costs limit the widespread clinical application to varying degrees. In recent years, the effectiveness of functional magnetic stimulation in the neurogenic bladder has gradually become apparent. Functional magnetic stimulation of the sacral nerve may be a promising treatment for neurogenic bladder after spinal cord injury, but there is currently a lack of research to confirm it. Therefore, a clinical randomized controlled trial is proposed to compare the efficacy of sacral nerve magnetism in stimulating the neurogenic bladder after spinal cord injury. We hypothesized that functional magnetic stimulation of the sacral nerve was effective in the treatment of neurogenic detrusor overactivity after spinal cord injury.

**Purpose of the study**

To evaluate the efficacy of repeated functional magnetic stimulation (rFMS) on the sacral nerve in patients with neurogenic detrusor hyperactivity (NDO) after suprasacral spinal cord injury (SCI) and to provide more options for rFMS treatment of NDO after suprasacral SCI.

**The research process**

1. **How many people will participate in this study?**

Approximately 31 people will participate in this study conducted by the Affiliated Hospital of Southwest Medical University, and about 31 people will participate in this study in this hospital.

1. **Research steps**

Before you are selected for the study, your doctor will ask, record your medical history, and perform a physical examination and magnetic resonance imaging of the spinal cord.

Once you have been determined that you can participate in this study, you will be randomly divided into rFMS and sham groups. The rFMS group will receive real rFMS treatment(magnetic spike stimulation scheme: 5Hz, 100% exercise threshold, once a day, 5 times a week for a total of 8 weeks) of the sacral nerve, while the sham group will receive sham stimulation. Both groups will receive similar treatment strategies, including medication, standard urine management, acupuncture treatment, and health evangelism. Before starting the intervention, your baseline data will be collected, as well as bladder capacity measured by urodynamic testing, the residual volume of the bladder, maximum Pdet, quality of life, bladder diary, NBSS and pudendal nerve electromyography. After that, interventions will be performed five times a week for 8 consecutive weeks. The bladder capacity, maximum detrusor pressure (Pdet) and pudendal nerve electromyography will be evaluated at baseline, 8th week of treatment. The residual volume of the bladder and bladder diary will be recorded once a week during 8 weeks of treatments. The Spinal Cord Injury – Quality of Life (SCI-QOL) and the Neurogenic Bladder Symptom Score (NBSS) will be evaluated at baseline, the 4th and 8th week of treatment. In addition, the above assessments will be followed up at 8 weeks after the end of treatment.

1. **How long will this study last?**

The study will last for 16 months. You can opt out of the study at any time without losing any benefit you would have received. However, if you decide to withdraw from this study enroute, we encourage you to consult with your doctor first. If you have a serious adverse event, or if your research doctor feels it is not in your best interest to continue participating in the study, he/she will decide to let it go You exit the study. Sponsors or regulators may also terminate the study for the duration of the study. However, your withdrawal will not affect your normal medical treatment and benefits.

If you quit the study for any reason, you may be asked about your participation in the study. You may also be asked to have laboratory tests and physical examinations if your doctor deems it necessary.

**4. Information and biological specimens collected in the study**

Before starting the intervention, participant baseline data will be collected, as well as bladder capacity measured by urodynamic testing, the residual volume of the bladder, maximum Pdet, quality of life, bladder diary, NBSS and pudendal nerve electromyography. After that, interventions will be performed five times a week for 8 consecutive weeks. The bladder capacity, maximum detrusor pressure (Pdet) and pudendal nerve electromyography will be evaluated at baseline, 8th week of treatment. The residual volume of the bladder and bladder diary will be recorded once a week during 8 weeks of treatments. The Spinal Cord Injury – Quality of Life (SCI-QOL) and the Neurogenic Bladder Symptom Score (NBSS) will be evaluated at baseline, the 4th and 8th week of treatment. In addition, the above assessments will be followed up at 8 weeks after the end of treatment.**Risks and benefits**

**1. What are the risks of participating in this study?**

The risks you may pose to participating in this study are as follows. You should discuss these risks with your research doctor, or if you prefer, with the doctor who usually cares for you.

During the study, you may experience some, all, or no of these adverse events, risks, discomforts, inconveniences such as headaches, scalp pain and discomfort, and facial twitch sensations, which are usually mild and do not affect the patient's life. The magnetic pulse produces a slight noise, the subject is usually not significantly disturbed, and the subject can also bring his own earplugs.

Acupuncture may present with the risks of needle sickness, bleeding, hematoma, etc., generally no serious adverse reactions, and the vast majority of risks are transient and reversible, which can be alleviated or eliminated by terminating training and resting.

At the same time, there may be risks in terms of information security. We do our best to protect the information you provide from disclosure, however, we cannot guarantee the absolute security of the information. You can rest at any time during the research process. You can withdraw from this study at any point in your study.

If you develop any discomfort during the study, or a new change in your condition, or any unexpected circumstances, whether or not related to the study, you should promptly notify your doctor, who will judge and give appropriate medical treatment.

You will need to be in the hospital on time during your studies to follow up and do some tests, which will take up some of your time and may also cause you trouble or inconvenience.

**2. What are the benefits of participating in the study?**

Direct benefits: If you agree to participate in this study, you may be able to receive direct medical benefits. This study will likely improve the degree of your neurogenic bladder and reduce disruption to your daily routine.

Potential benefits: This study may improve your quality of life. We hope that the information we receive from this study in which you participated will benefit you or patients with the same condition as you in the future.

**Alternative treatment options**

In addition to participating in this study, you can receive routine treatment from your doctor:

Indwelling catheterization

Discuss these and other possible options with your doctor.

**Use of research results and confidentiality of personal information**

With the understanding and assistance of you and other subjects, the results of research through this program may be published in medical journals, but we will keep your research records confidential as required by law. The personal information of the research subjects will be kept strictly confidential and your personal information will not be disclosed unless required by relevant laws. When necessary, government administrations, hospital ethics committees and other relevant researchers may consult your information as required.

**Regarding research expenses** **and related compensation**

**1. Cost of the drug/device used in the study and related examinations**

The daily urination frequency, the number of self-directed urinations, the amount of spontaneous urination, the maximum urinary flow rate, the residual urine output, the maximum detrusor pressure, the maximum safe capacity of the bladder, the pudendal nerve evoked potential, and the basic dataset of the international quality of life of spinal cord injury in this study are free items; rFMS and acupuncture as charged items; Routine treatment and testing for other diseases that you are also combining with will not be covered free of charge.

**2. Compensation** **for participation in the study**

There is no additional compensation for this study.

**3. Compensation/indemnification** **after the occurrence of damage**

If an injury related to this study occurs, you can receive free treatment provided by the Rehabilitation Department of the Affiliated Hospital of Southwest Medical University, or compensate/compensate according to the relevant laws of China.

**The rights of the subject and related precautions**

**1. Your Rights**

Throughout the process of participating in the study, you are voluntary. If you decide not to participate in this study, it will not affect the other treatments you should receive. If you decide to participate, you will be asked to sign this written informed consent form. You have the right to withdraw from the Trial at any stage of the Trial without discrimination or unfair treatment, and your medical treatment and rights will not be affected.

**2. Precautions**

As a subject, you need to provide real information about your medical history and current physical condition; Tell the study physician about any discomforts he or she found during this study; Do not take restricted medications, food, etc. that have been notified by your doctor; Tell the study doctor if he or she has been involved in other studies recently, or is currently participating in other studies.

**Contact information for obtaining information**

If there is any important new information during the course of the study that may affect your willingness to continue with the study, your doctor will notify you in a timely manner. If you are on your own research data, or if you would like to know the findings of this study after the study is over. You can ask any questions about this study at any time and get answers accordingly, please contact Maomao Huang by phone +86 18384308766.

The Ethics Committee has reviewed and approved the study, if you have any questions related to your rights/interests, or if you would like to reflect the difficulties, dissatisfaction and concerns encountered in participating in this study, or would like to provide comments and suggestions related to this study, please contact the Ethics Committee of the Affiliated Hospital of Southwest Medical University at 0830-3165273 / 3165972, Email: xnydfyirb@sina.com.

Subject signature page

Informed Consent Statement:

I have been informed of the purpose, background, process, risks and benefits of this study. I had plenty of time and opportunity to ask questions and I was satisfied with the responses.

I was also told who I should contact when I had questions, wanted to reflect difficulties, concerns, suggestions for research, or wanted further information or help with research.

I have read this informed consent form and agreed to participate in this study.

I know that I can choose not to participate in this study or withdraw from this study at any time during the study period without any reason*.*

I have known that if my condition is worse, or I have a serious adverse event, or if my research doctor feels it is not in my best interest to continue participating in the study, he/ She would decide to let me quit the study. Without my consent, funders or regulators may also terminate the study for the duration of the study. If this happens, the doctor will notify me promptly and the research doctor will discuss my other options with me.

I will be given a copy of this informed consent form containing my signatures and those of the researchers.

Subject Signature: Date:

(Note: If the subject is incapacitated/incapacitated, the signature and date of signature of the legal representative are required).

Signature by legal representative: Date:

(Note: If the subject is unable to read the informed consent form, an independent witness is required to prove that the researcher has informed the subject of all the contents of the informed consent form, and the independent witness needs to sign and sign the date).

Independent Witness Signature: Date:

Investigator Signature: Date:
